# Pseudo-Volkmann Contracture: A Case Report

**DOI:** 10.7759/cureus.57607

**Published:** 2024-04-04

**Authors:** Saab Mestarihi, Ahmad Almigdad, Kamal Hurani, Saad Haddad, Khalid BaniMelhem

**Affiliations:** 1 Department of Orthopedics, Royal Medical Services, Amman, JOR; 2 Department of Orthopedics, Prince Hamza Hospital, Amman, JOR

**Keywords:** contracture., flexor digitorum profundus, flexor tendons entrapment, volkmann contracture, pseudo-volkmann contracture

## Abstract

Pseudo-Volkmann contracture, also known as entrapment of the flexor muscles, occurs due to mechanical entrapment of the flexor myotendinous units in the fracture or adhesions after both bone forearm fractures. It differs from Volkmann contracture in that there is no ischemia or compartment syndrome, and there is an absence of muscle fibrosis intra-operatively. Patients with pseudo-Volkmann contracture typically present with an inability to extend the fingers passively or actively when the wrist is in extension. However, finger extension is possible when the wrist is flexed.

We present the case of a 16-year-old female who developed pseudo-Volkmann contracture subsequent to sustaining a both bone forearm fracture at the age of 4. Despite early recognition of finger flexion issues, the diagnosis was delayed until the age of 16 due to parental reassurance. Consequently, further medical consultation was not sought as the child exhibited satisfactory functional abilities. Understanding these complications is crucial for administering appropriate treatment and mitigating the risk of long-term issues such as flexion contracture.

## Introduction

Pseudo-Volkmann contracture, also known as entrapment of the flexor muscles in the literature, occurs due to mechanical entrapment of the flexor myotendinous units in the fracture or adhesions that complicate both bone forearm fractures [1]. It should be noted that Pseudo-Volkmann contracture differs from Volkmann contracture, which arises as a complication of ischemia or compartment syndrome [2]. Clinical differentiation between the two syndromes relies on the presence of ischemic symptoms, such as disproportionate pain at the time of injury or treatment and, intraoperatively, the presence of ischemic muscle in Volkmann contracture. However, in pseudo-Volkmann contracture, there is no ischemia or compartment syndrome, and intraoperatively, there is no muscle fibrosis; instead, the main findings involve muscle entrapment or adhesions [3]. Patients with pseudo-Volkmann contracture typically present with an inability to extend the fingers passively or actively when the wrist is in extension. However, finger extension is possible when the wrist is flexed [4]. This clinical presentation also resembles mild cases of Volkmann contracture [5]. Early treatment of pseudo-Volkmann contracture usually results in a good prognosis, whereas delayed treatment may lead to joint contractures and less favorable outcomes [6].

We present the case of a 16-year-old female who developed pseudo-Volkmann contracture subsequent to sustaining a both bone forearm fracture at the age of 4. Despite early recognition of finger flexion issues, the diagnosis was delayed until the age of 16 due to parental reassurance. Consequently, further medical consultation was not sought as the child exhibited satisfactory functional abilities.

## Case presentation

A 16-year-old right-handed female sustained a closed fracture of the left forearm radius and ulna at the age of 4, which was managed conservatively. Throughout her presentation and treatment period, her pain was well-controlled, and there were no signs of compartment syndrome or neurological deficits. She received treatment with an above-elbow cast. At two weeks’ post-fracture, her parents noticed that her fingers began to adopt a bending position, primarily affecting the ring finger, followed by the middle and little fingers, but they were reassured regarding this observation. Eventually, the fracture healed, and the cast was removed at six weeks. The child was subsequently referred to physiotherapy, and a nerve conduction study was conducted to exclude nerve injury as a cause for the fingers' flexion position, which returned normal results. The patient and her parents were reassured and informed about the potential need for tendon lengthening after puberty. Consequently, they did not seek further medical assistance until the age of 16. Upon assessment by a general orthopedic surgeon, another nerve conduction study was conducted, which also yielded normal results. Subsequently, the patient was referred to an upper limb clinic.

**Figure 1:**
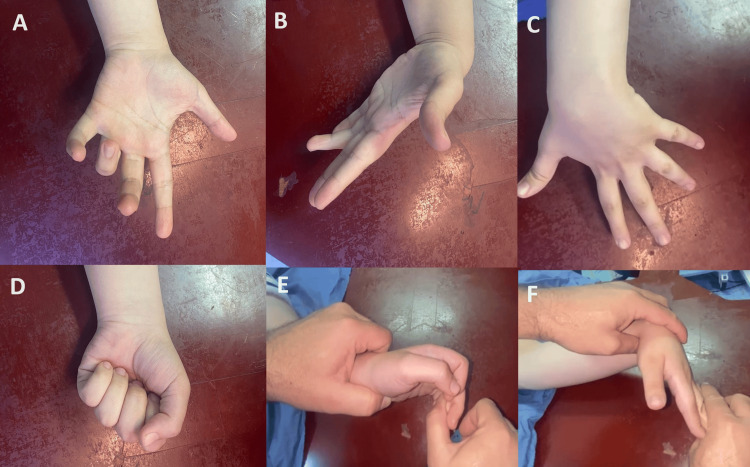
Preoperative clinical features. (A) flexion of middle-little fingers while wrist in extension. (B) Deformity corrected at the middle and little fingers while the wrist is flexed but remains in the ring finger. (C) Wrist is ulnarly deviated with finger extension. (D) Normal flexion of fingers. (E) No passive extension of the middle and little fingers when the wrist is extended. (F) Fingers can be extended when the wrist is flexed.

**Figure 2:**
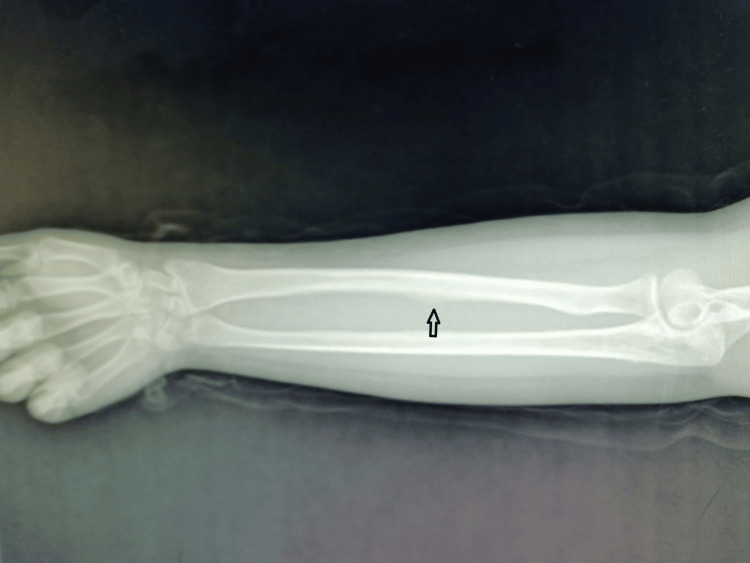
The anteroposterior radiograph of the radius and ulna reveals characteristics consistent with a healed fracture of the ulna (indicated by the arrow).

Clinical examination revealed that the patient could not extend her middle, ring, and little fingers at the proximal interphalangeal joint when the wrist was in a neutral or extended position. However, she could extend their distal interphalangeal joints. Complete extension and flexion of the fingers were possible when the wrist was flexed. The wrist deviated ulnarly and flexed when she extended her fingers (Figure 1). The ring finger was most severely affected, with limited extension beyond 90° when the wrist was extended. The middle finger was affected to a lesser extent. Neurovascular examination yielded normal results. Radiographic evaluation showed a healed fracture at the mid-ulna shaft (Figure 2). Consequently, entrapment of the flexor digitorum profundus (FDP) of the middle to little fingers was suspected

**Figure 3:**
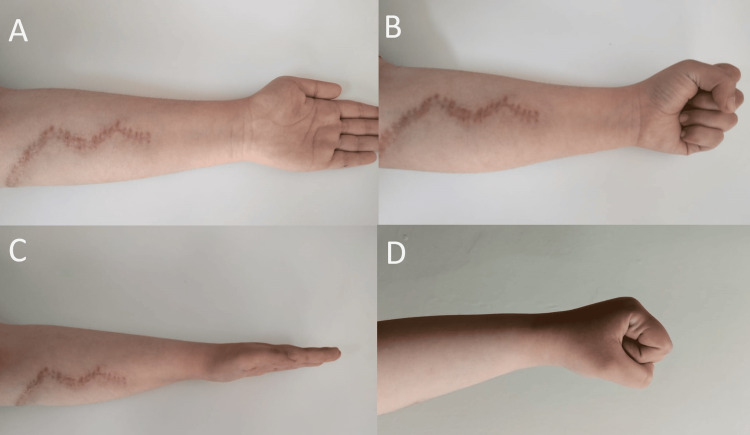
Post-operative finding. Note the zigzag incision over the proximal and middle volar forearm, which was used for flexor digitorum profundus release. (A-D) Normal wrist and fingers’ range of movements.

At surgery, the proximal and middle volar forearms were explored via a zigzag incision, and the median and ulnar nerves were identified and protected. Muscles were found to be viable. However, there were features indicative of an old healed fracture at the mid-shaft of the ulna, with adhesions of the FDP at the fracture site and interosseous membrane. The release of the adhesions at the muscle and fracture allowed immediate full passive extension of the middle to little fingers, with the wrist in flexion or extension (Figure 3). Active and passive range-of-motion exercises were initiated immediately after surgery to prevent the recurrence of adhesions.

## Discussion

Pseudo-Volkmann contracture, arising from flexor tendon entrapment or adhesions, was historically considered rare following pediatric forearm fractures. However, recent recognition of pseudo-Volkmann contracture suggests it may not be as rare as previously believed [7]. High-energy trauma is often implicated [8]. Mechanisms include entrapment of the muscle belly or muscle fibrosis secondary to hemorrhage at the fracture site [3]. Anatomical reduction of both bones during manipulation can help prevent this complication. Skeletally mature both bone forearm fractures are usually treated with open reduction to minimize this risk, although adhesions remain a concern [9]. Careful examination of digital joint motion before and after manipulation is crucial for diagnosis. Immobilization of the wrist can delay diagnosis, as this complication may not be immediately recognized. Delay in diagnosis may also be attributed to the isolated entrapment of the flexor digitorum superficialis [10].

Clinical findings of pseudo-Volkmann contracture include the inability to extend fingers passively or actively when the wrist is in extension, but full extension is possible when the wrist is flexed [4]. Unlike Volkmann ischemic contracture, there are no ischemic clinical signs or ischemic muscle found during surgical treatment [5]. Although extensor tendons can be similarly entrapped, this does not mimic Volkmann ischemic contracture [11].

Delay in diagnosis is common, but surgical release can lead to full or near-full recovery, even in cases presenting years after the injury [5]. Pseudo-Volkmann contracture often results from entrapment or adhesion of the FDP on the ulna, particularly associated with a short, oblique ulna fracture pattern. The ring finger is commonly affected due to its anatomical proximity to the ulna [1]. However, entrapment can also occur at a distal radius fracture [12].

In our case, the contracture developed after two weeks following the injury. However, unawareness of the condition led to delayed treatment until 12 years later. The fact that the nerve conduction test was conducted twice suggests that the treating doctors suspected nerve problems. Additionally, the parents reported that they were informed about tendon lengthening after puberty, indicating that their doctor suspected Volkmann contracture. The flexion deformity of the fingers was flexible, and the longstanding condition did not progress to a rigid contracture. The child adapted to her condition, and consequently, the parents did not seek medical help until the age of 16, primarily due to concerns about cosmetic appearance.

Based on the above, the delayed recognition and treatment of the child's condition were primarily due to a lack of awareness among orthopedic surgeons about such complications, highlighting its rarity. Therefore, orthopedic surgeons should be aware of this potential complication that might occur after forearm both bone fractures.

## Conclusions

The delayed recognition and treatment of pseudo-Volkmann contracture underscore the importance of increased awareness among orthopedic surgeons regarding such potential complications following forearm fractures. Early identification and intervention can significantly improve patient outcomes. Therefore, it is essential for clinicians to remain vigilant for this rare but potentially debilitating complication in patients with forearm fractures.
